# Duration of Intrapartum Antibiotics for Group B Streptococcus on the Diagnosis of Clinical Neonatal Sepsis

**DOI:** 10.1155/2013/525878

**Published:** 2013-03-28

**Authors:** Mark A. Turrentine, Anthony J. Greisinger, Kimberly S. Brown, Oscar A. Wehmanen, Melanie E. Mouzoon

**Affiliations:** ^1^Department of Obstetrics & Gynecology, Kelsey-Seybold West Clinic, 1111 Augusta Drive, Houston, TX 77057, USA; ^2^Kelsey Research Foundation, 5615 Kirby Drive, Suite 660, Houston, TX 77005, USA; ^3^Department of Pediatrics, Kelsey-Seybold Clinic, Kelsey-Seybold Main Campus, 2727 West Holcombe Boulevard, Houston, TX 77025, USA

## Abstract

*Background*. Infants born to mothers who are colonized with group B streptococcus (GBS) but received <4 hours of intrapartum antibiotic prophylaxis (IAP) are at-risk for presenting later with sepsis. We assessed if <4 hours of maternal IAP for GBS are associated with an increased incidence of clinical neonatal sepsis. *Materials and Methods*. A retrospective cohort study of women-infant dyads undergoing IAP for GBS at ≥37-week gestation who presented in labor from January 1, 2003 through December 31, 2007 was performed. Infants diagnosed with clinical sepsis by the duration of maternal IAP received (< or ≥4-hours duration) were determined. *Results*. More infants whose mothers received <4 hours of IAP were diagnosed with clinical sepsis, 13 of 1,149 (1.1%) versus 15 of 3,633 (0.4%), *P* = .03. Multivariate logistic regression analysis showed that treatment with ≥4 hours of IAP reduced the risk of infants being diagnosed with clinical sepsis by 65%, adjusted relative risk 0.35, CI 0.16–0.79, and *P* = .01. *Conclusion*. The rate of neonatal clinical sepsis is increased in newborns of GBS colonized mothers who receive <4 hours compared to ≥4 hours of IAP.

## 1. Introduction

Guidelines for group B streptococcus (GBS) originally established in 1996 (and reaffirmed in 2002 and 2010) by the Centers for Disease Control and Prevention (CDC) identify asymptomatic infants born to mothers who were colonized with GBS but received <4 hours of intrapartum antibiotics, as at-risk for presenting later with sepsis [1–3]. Past management recommendations for infants have been limited either to evaluation with a blood culture and complete blood count or more recently only just to observation for ≥48 hours [1–3]. The origin of this four-hour duration for intrapartum GBS antibiotic prophylaxis is unclear. In the presence of at least one risk factor such as premature delivery <37-week gestation, rupture of membranes >6 hours, or maternal fever of ≥37.5°C, intrapartum antibiotic prophylaxis of <4 hours results in higher rates of vertical transmission of neonatal GBS colonization [4, 5]. Yet even in the presence of maternal risk factors, if intrapartum antibiotic was given at least 2 hours before delivery, the effectiveness in preventing early onset group B streptococci disease was demonstrated [6]. However, the majority of infants exposed to GBS at birth are delivered to colonized mothers without additional risk factors. 

Small prospective observation studies in women without risk factors have shown a lower rate of infant colonization with GBS, if maternal intrapartum prophylaxis was >4-hour duration [7–9]. Yet in these studies no neonates whose mothers received any duration of intrapartum antibiotic prophylaxis developed GBS disease. Two of these trials looked specifically if shorter durations of intrapartum antibiotics were of benefit and noted that if mothers received at least 2 hours of antibiotic therapy, rates of infant colonization were reduced when compared to no treatment [7, 9]. However, a systematic review of the medical literature did not find evidence that those infants whose mothers had <4 hours of intrapartum prophylaxis for GBS were at any higher risk for sepsis than those infants born after ≥4 hours of exposure [10]. It was suggested that continued monitoring of the influence of GBS prevention recommendations on the management of newborns was needed [3]. The objective of this study was to determine whether <4 hours of maternal antibiotic prophylaxis for GBS, without other risk factors, increase the diagnosis of clinical neonatal sepsis.

## 2. Materials and Methods

This is a retrospective cohort study of women-infant dyads undergoing intrapartum antibiotic prophylaxis for group B streptococcus at ≥37 0/7 weeks of estimated gestational age (EGA) who presented in labor with a planned vaginal delivery. The Woman's Hospital of Texas is a community hospital in Houston, TX, USA, with private physicians. The hospital averages more than 8,000 deliveries per year and has 350 pediatricians on staff. The hospital has a level III neonatal intensive care unit (NICU) staffed by 12 neonatologists. A computerized search of medical records dated from January 1, 2003 through December 31, 2007 was performed to identify mothers undergoing antibiotic prophylaxis for GBS. Any potential cases of mothers or infants that were treated for GBS were identified by utilizing the *International Classification of Diseases*, 9th Revision Clinical Modification codes in hospital discharge summaries as follows: GBS carrier or suspected carrier (V02.51); infections of genitourinary tract in pregnancy (646.6x); unspecified infectious conditions in the mother (647.94); other current conditions in mother complicating pregnancy (647.81); maternal conditions affecting the fetus or newborn (760.2 and 760.8); other infections specific to the perinatal period (771.89); observation and evaluation of newborns for suspected infectious condition (V29.0); and septicemia of newborn (771.81) [11]. Medical records were then reviewed to confirm maternal cases that were treated for intrapartum GBS and the indication for therapy. This study was approved by the institutional review board at The Woman's Hospital of Texas. 

Inclusion criteria included women undergoing intrapartum antibiotic prophylaxis for GBS colonization. During the study period, the 2002 CDC guidelines for a culture-based screening strategy to detect GBS status at 35 to 37 weeks of EGA with implementation of chemoprophylaxis for prevention of early onset GBS disease in the newborn were followed [2]. All singleton live births with planned vaginal delivery at ≥37 0/7 weeks of gestation were included. To eliminate any bias of treatment for the risk for early onset disease due to early gestational age, only pregnancies that delivered at ≥37 0/7 weeks of gestation were evaluated. Exclusion criteria included scheduled cesarean delivery or the development of chorioamnionitis. Medical records were reviewed by trained research nurses and variables extracted included: maternal age and race, estimated gestational age at delivery, type and duration of intrapartum antibiotic therapy, duration of rupture of membranes, and mode of delivery. Fetal outcome included the number of infants stratified by the duration of intrapartum maternal antibiotics received (either < or ≥4-hour duration) and the number of infants labeled with the discharge diagnosis of clinical sepsis. A planned secondary analysis of the diagnosis of neonatal clinical sepsis was based on the duration of maternal intrapartum antibiotic prophylaxis as follows: <2 hours, 2 to <4 hours, and ≥4 hours. An abnormal CBC was defined as a total white blood cell count (WBC) of ≤5000 or ≥30,000/mm [12]. Infants with signs and symptoms consistent with early onset sepsis were evaluated by a neonatologist. Infants were then transferred to a higher level of neonatal care for intravenous antibiotic therapy, either an intermediate level nursery or NICU (if cardiovascular or respiratory support was required). The infant would have a CBC and blood culture obtained (if not already performed) and begun on intravenous broad spectrum antibiotics (ampicillin and gentamicin) for a minimum of 48 hours and until the blood culture was negative. Chest radiograph was performed if the neonate had respiratory symptoms. Lumbar puncture was performed at the discretion of the neonatologist. Newborns diagnosed with clinical sepsis had their records reviewed to corroborate if the following clinical signs suggestive of early onset sepsis were present: fever (>38.0°C); hypothermia (<36.5°C); lethargy; tachypnea (respiratory rate > 60 breaths per minute); apnea (cessation of respiration for >20 seconds); bradycardia (<100 beats per minute); cyanosis; and hypoglycemia (glucose < 60 mg/dL and not due to other diagnosis) [12, 13]. Infants who had a positive blood or cerebral spinal fluid culture result and had clinical signs of infection were classified as septic. Infants with a positive blood culture result and no clinical signs of infection were classified as bacteremic. Infants were classified as having clinically suspected GBS infection if there were two or more clinical signs of infection but negative cultures from a sterile site, and their mothers had positive intrapartum culture results for GBS [13]. Clinical sepsis was defined as the total number of infants that were septic and/or had a clinically suspected GBS infection [13, 14].

Among women with an indication for GBS prophylaxis, it has been reported that 71% will receive optimal chemoprophylaxis (defined as initiation of a recommended antibiotic 4 hours or more before delivery) [15]. We therefore assumed that for every infant that received <4 hours of intrapartum antibiotic prophylaxis (the study group), two infants would receive ≥4 hours of adequate intrapartum antibiotic prophylaxis (the control group). It has been shown that in asymptomatic at-risk newborns, approximately 1% develops early onset sepsis [12]. The efficacy of intrapartum antibiotics in preventing early onset group B streptococcal disease in infants of culture-positive women without clinical risk factors has been shown to be greater than 80% [14, 16]. If one assumes a 1% risk of developing clinical sepsis in term asymptomatic at-risk newborns (i.e., those that receive <4 hours of intrapartum antibiotics) [12], then to detect an 80% reduction in the development of clinical sepsis (1% to 0.2%), with two controls to each study patient at *P* < .05 and a power of 80%, would require 988 infants in the study group and 1976 in the control group. 

Univariate analyses were conducted to determine whether there were significant differences between mothers and infants that received < versus ≥4 hours of intrapartum antibiotic prophylaxis with respect to each variable present. For categorical variables, the *χ* ^2^ test was used and the paired *t* test for continuous measures. In multivariate analysis, odds ratios (OR) were assumed to approximate relative risks (RR) because clinical sepsis was rare in this population [16]. Multivariate logistic regression analysis was performed on independent variables with a *P* < .10 to estimate the adjusted OR with 95% confidence interval (CI) to control for factors that would affect the rate of clinical diagnosis of sepsis. *P* < .05 was considered statistically significant. Statistical analysis was performed using STATA, release 11 (Stata Corp, College Station, TX, USA).

## 3. Results

**Figure 1:**
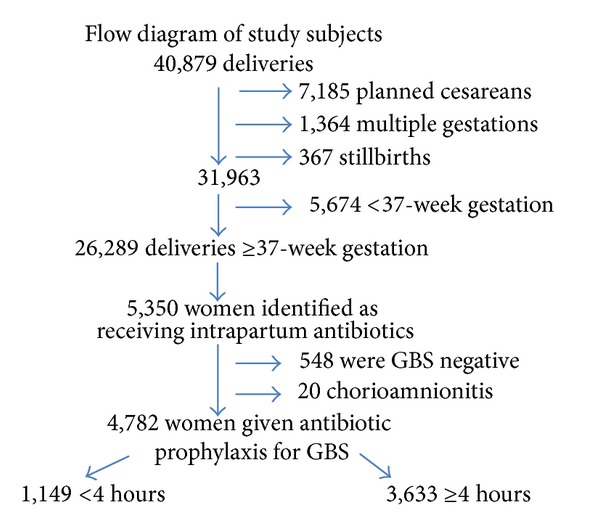
Study population.

During the five-year study period, 40,879 deliveries greater than 20-week gestation occurred. Of the 26,289 eligible deliveries, 5,350 (20.4%) were identified as being potentially treated with intrapartum antibiotics for GBS prophylaxis. Medical record review provided a group of 4,782 women available for analysis. A flow chart is shown in Figure 1. The majority of women, 4,756 (99%), received antibiotic prophylaxis ranging from 2 minutes to 44.4 hours, with penicillin (84.9%) being the most common agent utilized. Other antibiotics utilized were cefazolin (5%), ampicillin (4%), clindamycin (3%), vancomycin (1%), erythromycin (0.02%), and other (2%). The most common indications for antibiotic prophylaxis were a positive vaginal-rectal culture for GBS (72.1%) or a urine culture with GBS bacteriuria (12.1%). The remaining indications for antibiotic administration were order by physician for GBS prophylaxis but no documentation of GBS result (9.9%); colonization in a previous pregnancy (5.3%); previous infant with GBS sepsis (0.4%); and patient reporting they were GBS colonized but no documentation available in the medical chart (0.2%). The majority of women underwent vaginal delivery (80%).

Women were divided into two groups based on duration of antibiotic prophylaxis with 1,149 receiving <4 hours and 3,633 receiving ≥4 hours. Characteristics of women and neonates by duration of antibiotic prophylaxis are shown in Table 1. Mothers who received ≥4 hours of intrapartum antibiotics were slightly younger than those who received <4 hours of antibiotics (29.4 versus 30.1 years, *P* < .01); otherwise, no other maternal differences were noted. Of the infants who received <4 hours of antibiotic prophylaxis for maternal colonization with GBS, 818 (71%) had a CBC and 777 (68%) a blood culture performed. A greater number of infants whose mothers received <4 hours of antibiotic prophylaxis for GBS colonization were admitted to the NICU, 11 of 1,149 (0.96%) compared to ≥4 hours of antibiotic prophylaxis, 15 of 3,633 (0.41%), *P* = .04. The, overall length of infant hospital stay was not different between groups, 2.6 days versus 2.5 days for < or ≥4 hours of intrapartum antibiotic therapy, *P* = .99. When only mothers who underwent vaginal delivery were evaluated, the overall length of infant stay was longer for infants whose mothers received <4 hours of antibiotic prophylaxis compared to ≥4 hours. However, this difference was not statistically significant, 2.46 versus 2.25 days, *P* = .07, respectively. When infants that had the clinical diagnosis of sepsis were excluded, regardless of the mode of delivery, the length of infant stay did not change (data not shown). 

A larger number of infants whose mothers received <4 hours of antibiotic prophylaxis (compared to ≥4 hours) were given the discharge diagnosis of clinical sepsis 13 of 1,149 (1.1%) versus 15 of 3,633 (0.4%), *P* = .03. When analysis was limited to mothers colonized with GBS by vaginal-rectal culture and urine (*n* = 4,028), similar findings were noted, <4 hours of antibiotic prophylaxis compared to ≥4 hours, and were given the discharge diagnosis of clinical sepsis, 13 of 917 (1.4%) versus 13 of 3,111 (0.4%), *P* < .01. In mothers who received <4 hours of intrapartum antibiotic prophylaxis, 120 (14.7%) of infants had an abnormal WBC count and 5 (0.64%) a positive blood culture. Three infant's blood cultures were suspected skin contaminates (coagulase negative staphylococcus). The remaining two infant's blood cultures grew GBS. In mothers who received ≥4 hours of intrapartum antibiotic prophylaxis, no infant had an abnormal WBC count, and 1 (0.03%) infant had a positive blood culture for Bacillus cereus. Seventy-five percent of infants with the diagnosis of clinical sepsis were symptomatic within the first 6 hours of life, and all were by 24 hours. All infants diagnosed with clinical sepsis had at least two clinical signs suggestive of early onset sepsis and 61% had three or more. 

To determine the independent association between the development of neonatal clinical sepsis with duration of intrapartum antibiotic treatment, multivariate logistic regression analysis was performed including significant maternal intrapartum factors identified in the univariate analysis (Table 1). When adjusted for maternal age and the duration of rupture of membranes, treatment with ≥4 hours of intrapartum antibiotics reduced the risk of infants being diagnosed with clinical sepsis by 65%, adjusted relative risk 0.35, CI 0.16–0.79, and *P* = .01. The duration of intrapartum antibiotic administration impacted the diagnosis of neonatal clinical sepsis; with the diagnosis of neonatal sepsis decreasing the longer the mother received intrapartum antibiotics: 1.6% for <2 hours, 0.9% for 2 to <4 hours, and 0.4% for ≥4 hours (Table 2). Infants whose mothers received <2 hours of intrapartum antibiotic prophylaxis had the greatest risk of being diagnosed with clinical sepsis, adjusted relative risk 3.5, CI 1.3–9.6, and *P* = .015.

## 4. Discussion

This study demonstrated that in asymptomatic at-risk term newborns exposed to <4 hours of intrapartum antibiotic prophylaxis for GBS, a greater percentage will be transferred to a higher level of nursery care and will ultimately be given the discharge diagnosis of clinical sepsis. Further, the duration of intrapartum antibiotic administration impacted the diagnosis of neonatal clinical sepsis; with the diagnosis of neonatal sepsis decreasing the longer the mother received intrapartum antibiotics. Infants whose mothers received <2 hours of intrapartum antibiotic prophylaxis had the greatest risk of being diagnosed with clinical sepsis.

Previous studies assessing the optimal timing of intrapartum antibiotic prophylaxis for maternal colonization with GBS, without other risk factors, have shown increased rates of neonatal GBS colonization but no increased risk of neonatal sepsis in infants whose mothers received <4 hours of antibiotic therapy [7–9]. Due to the rare incidence of neonatal GBS sepsis, these small studies relied on neonatal colonization as a surrogate marker for neonatal GBS sepsis. In the largest study, 137 neonates whose GBS colonized mothers received <4 hours of intrapartum antibiotics demonstrated a colonization rate of 3.6%, which was statistically less (*P* < .001) than the 60% rate of colonization in a control group of 30 neonates whose mothers did not receive treatment [9]. No differences were noted in neonatal GBS colonization rates in ≤2 hours (2.4%) versus >2 but <4 hours (5.5%) of antibiotic therapy. Infants from mothers who had received adequate intrapartum antibiotic therapy (i.e., ≥4 hours) were excluded from the analysis. Other researchers have noted a significant reduction in the number of GBS colonized newborns whose mothers received at least 4 hours of intrapartum antibiotic prophylaxis. Lijoi et al. [8] noted that 3.7% of 136 infants whose mothers received ≥4 hours of intrapartum antibiotic was significantly reduced (*P* < .001) compared to the 12.3% of the 73 infants whose mothers had <4 hours of treatment. However, they did not have enough sample size to evaluate the benefit of intrapartum antibiotic prophylaxis for shorter durations of therapy. The only previous study demonstrating a difference in the duration of intrapartum antibiotic therapy on the effect of neonatal GBS colonization was by de Cueto et al. [7]. They noted that in 115 women who received <4 hours of intrapartum antibiotic prophylaxis, when maternal antibiotic prophylaxis was >2 hours, the newborn colonization rate (2.9%) approached the rate seen in infants whose mothers received >4 hours of therapy (1.2%). However, this study had some limitations in that intrapartum antibiotic therapy was administered 12 hours after the start of labor (the time required for a rapid GBS test result to be available) and that 43% of mothers had at least one risk factor for GBS disease [17]. The findings of our study suggest that women colonized with GBS, without other risk factors, whose infants receive <4 hours of intrapartum antibiotic prophylaxis are at greater risk of GBS disease compared to those newborns whose mothers were given ≥4 hours of therapy. Infants that received <4 hours of intrapartum antibiotic prophylaxis for maternal GBS colonization carried over a twofold increase in risk for the clinical diagnosis of neonatal sepsis. 

When the duration of maternal intrapartum antibiotic was correlated with neonatal clinical outcomes, a higher rate of the diagnosis of clinical sepsis was noted in infants whose mothers received <2 hours of antibiotic prophylaxis. This observation is in accord with studies from women colonized with GBS with intrapartum risk factors that durations of intrapartum antibiotic prophylaxis of at least 2 hours may have some benefit [5, 6]. In a small prospective observation trial evaluating the effects of intrapartum antibiotic prophylaxis in 70 GBS positive mothers with intrapartum risk factors, the only cases of GBS sepsis were in infants whose mothers received ≤2.5 hours of antibiotic prophylaxis [5]. Further, in a case-control study evaluating the effectiveness of risk-based intrapartum antibiotic prophylaxis for GBS sepsis, if intrapartum antibiotic prophylaxis was given ≥2 hours before delivery, the effectiveness for prevention of GBS disease was 89% [6]. In the present study, the two infants diagnosed with GBS sepsis had mothers who received less than 2 hours of intrapartum antibiotic prophylaxis. 

Pharmacokinetic studies of *β*-lactam antibiotics utilized for maternal prophylaxis for intrapartum GBS would suggest that bactericidal levels in fetal blood are achieved as early as 3 minutes with higher levels persisting for up to 2 hours [18–20]. Why then do not durations of <2 hours of maternal intrapartum antibiotic prophylaxis reduce neonatal GBS disease equivalent to longer antibiotic durations? It has been suggested that cases of neonatal GBS sepsis that occur with short durations of maternal antibiotic prophylaxis may represent fetal exposure to GBS in utero prior to antibiotic administration when tissue injury by GBS may not be quickly reversible [18, 20]. What minimal duration of intrapartum antibiotic therapy for GBS prophylaxis is adequate to prevent neonatal GBS disease cannot be determined from the current study. Although no difference was noted in the number of cases of infant clinical sepsis whose mothers received 2 hours but <4 hours compared to adequate duration of intrapartum antibiotics (i.e., ≥4 hours), there was insufficient numbers to rule out a type II error.

Several limitations in our study should be considered. The study is retrospective and relied on an administrative database (i.e., ICD-9 codes) to identify women and infants treated for GBS colonization. Since, a standardized definition of clinical sepsis was not predefined, this finding must be interpreted with caution. Selection bias may have played a role in these results. Since by definition, infants that received <4 hours of intrapartum antibiotic prophylaxis were considered “at-risk,” the physicians involved in their care may have had a lower clinical threshold to monitor these infants for closer evaluation and ultimately label them with the diagnosis of sepsis. The rate of neonatal clinical sepsis in our study in this at-risk group was similar to the 1% rate noted in previous studies [12]. Further, review of infant medical records that were diagnosed with clinical sepsis confirmed diagnostic criteria utilized in previous reports, although past studies may have similar risks of bias [12–14]. Although we would have expected symptomatic newborns to have returned for readmission to the pediatric unit at the study hospital, it is possible that some infants who became ill after discharge could have returned to another area hospital for treatment. Finally, the extrapolations of the results of the current study are limited since it was conducted at one institution in a single city.

On the other hand, the strengths of this study warrant attention. Women who were GBS colonized without other risk factors were exclusively evaluated, which represents the majority of GBS positive mothers. A large number of asymptomatic, at-risk infants were managed by 350 pediatricians which may allow for a more generalizability of these study results. The cohort study design is less prone to selection bias and residual confounding and the effect size of the association of ≥4 hours of intrapartum antibiotic therapy on reducing neonatal clinical sepsis (adjusted relative risk 0.35) indicates a range that merits further consideration [21]. 

## 5. Conclusions

We examined a large cohort of women who were GBS carriers without other risk factors. It appears that infants whose mothers receive <4 hours of intrapartum antibiotic prophylaxis for GBS colonization are at an increased risk for being diagnosed with clinical sepsis. Larger studies are needed to determine whether shorter specific durations of maternal antibiotic prophylaxis will reduce this risk.

## Figures and Tables

**Table 1:** Frequency of maternal and neonatal characteristics by the duration of exposure to intrapartum antibiotic therapy for group B streptococcus.

	<4 hours of antibiotics	≥4 hours of antibiotics	*P* value
	*N* = 1,149	*N* = 3,633	
Maternal age (years)	30.1 ± 6.2	29.4 ± 7.4	<.01
Race			.20
White	547 (48)	1,828 (50)	
Black	233 (20)	730 (20)	
Hispanic	251 (22)	698 (19)	
Asian	64 (6)	167 (5)	
Other	54 (5)	210 (6)	
EGA at delivery (weeks)	39.0 + 1.0	39.1 + 1.0	1.0
Duration of ROM (hours)	2.8 ± 3.2	6.8 ± 5.7	<.01
% with ≥18 hours of ROM	0	11 (0.3)	
Duration of antibiotics (hours)	2.5 ± 1.1	8.8 ± 4.8	<.01
Birth weight (grams)	3,341 ± 454	3,404 ± 433	<.01
NICU nursery admission	11 (1)	15 (0.4)	.04
Length of infant stay	2.6 ± 4.0	2.5 ± 3.5	.99

Data are mean ± standard deviation or *n* (%).

EGA: estimated gestational age; ROM: rupture of membranes; NICU: neonatal intensive care unit.

**Table 2:** Effect of the duration of intrapartum antibiotic prophylaxis on the diagnosis of neonatal clinical sepsis.

Duration of IAP	Number of women	Diagnosis of neonatal clinical sepsis		
<2 hours	385	6 (1.6)		
2 to <4 hours	764	7 (0.9)		
≥4 hours	3,633	15 (0.4)		

	RR (95% CI)^#^			
	Crude	*P*	Adjusted*	*P *

<2 hour versus ≥4 hours	3.8 (1.5–9.7)	.01	3.5 (1.3–9.6)	.02
2 to <4 hours versus ≥4hours	2.2 (0.9–5.4)	.08	2.1 (0.8–5.5)	.12

IAP: intrapartum antibiotic prophylaxis; RR: relative risk; CI: 95th percentile confidence interval.

Data are *n* (%) unless otherwise specified.

^#^In multivariate analysis, odds ratios (OR) were assumed to approximate relative risks (RR) because clinical sepsis was rare in this population [16].

*Adjusted by multivariate logistic regression for maternal age and the duration of rupture of membranes.
